# Mouse pancreatic tumor organoids reveal synergistic efficacy of low-dose anticancer drug and radiation combinations

**DOI:** 10.3389/fmed.2025.1661521

**Published:** 2025-09-16

**Authors:** Zachery Keepers, Aniketh Sharma, Sanjit Roy, Hurley Ryan, Binny Bhandary, Lei Ren, Narottam Lamichhane, Hem D. Shukla

**Affiliations:** ^1^Department of Radiation Oncology, University of Maryland School of Medicine, Baltimore, MD, United States; ^2^Department of Biomedical Engineering, University of Illinois at Urbana-Champaign, Urbana, IL, United States

**Keywords:** pancreatic cancer, radiation therapy, organoid, combination therapy, 5-fluorouracil (5-FU), gemcitabine, ROS, double-stranded breaks (DSBs)

## Abstract

**Background:**

Pancreatic cancer is the fourth-leading cause of cancer death in the United States, with a 5-year survival rate of only 13%. Most patients with locally advanced pancreatic cancer receive chemotherapy with or without radiation therapy (RT). However, current treatment approaches often result in limited clinical response, highlighting the need for novel therapeutic strategies tested in robust model systems. Pancreas tumor-derived organoids offer a promising representative preclinical model for assessing responses to chemotherapy drugs, RT, and combination treatments.

**Methods:**

Pancreatic tumor organoids (PTOs) were derived from Panc02 mouse flank tumors. The PTO microenvironment was characterized and compared with the *in vivo* tumor using immunohistochemical and immunofluorescence staining for alpha-smooth muscle actin (α-SMA) and vimentin. The organoids were treated with fractionated x-ray radiation, gemcitabine, 5-fluorouracil (5-FU), and combinations of drugs with radiation. Treatment response was observed and quantified using brightfield imaging and immunofluorescence to detect reactive oxygen species (ROS) and γH2AX.

**Results:**

Three-dimensional PTOs exhibited expression patterns of α-SMA and vimentin similar to *in vivo* tumors, underscoring their relevance as a translational preclinical model. Dose-dependent growth suppression was observed following treatment with individual chemotherapy agents and radiation. Combination treatments with low-dose chemotherapy and radiation resulted in significantly greater inhibition of organoid growth compared to single-modality treatments. This enhanced effect was validated by reduced vimentin expression, increased γH2AX expression, and elevated reactive oxygen species (ROS) production, indicating amplified DNA damage and cytotoxicity.

**Conclusion:**

Combining low-dose chemotherapy with radiation is significantly more effective at inhibiting pancreatic tumor organoid growth than either treatment alone, likely by targeting distinct signaling pathways. Additionally, the tumor organoid model holds promise for examining drug and radiation treatment responses, with potential for translational impact.

## Introduction

Pancreatic cancer is the fourth leading cause of cancer-related deaths. In 2025, the estimated number of pancreatic cancer cases was 67,440, with 51,980 deaths reported (1). The high mortality rate is primarily attributed to late-stage diagnosis, tumor heterogeneity, and resistance to conventional treatments (2). Chemotherapy and radiation therapy remain standard treatment modalities; however, monotherapy often yields variable responses, underscoring the need for individualized treatment strategies (3, 4).

Pancreatic ductal adenocarcinoma (PDAC) has been extensively studied using various models, including two-dimensional cell lines and *in vivo* mouse models. However, these models have limitations and may only offer partial insight into treatment response (4). Two-dimensional cell line models fail to accurately simulate the complexity of the three-dimensional (3D) tumor microenvironment (TME). *In vivo* mouse models, while more representative, are time-consuming and costly. Therefore, in this study, we used a 3D tumor-derived organoid model, which more accurately mimics the diversity and architecture of a tumor, is cost-effective and generally quick to generate, and can be extensively propagated for experimental manipulation (5, 6).

Tumor organoids derived from pancreatic tissue have shown promise as translational models (7, 8). Additionally, 3D organoids mimic functional characteristics of tumors when transplanted into mice (9) and exhibit treatment responses similar to those of the original tumor (3, 10). They also retain the expression of stromal and epithelial markers, enabling study of the response of the TME to different therapies (11).

Standard therapies for PDAC include FOLFIRINOX, which is a combination of 5-fluorouracil (5-FU), oxaliplatin, irinotecan, and leucovorin, or gemcitabine and nab-paclitaxel with or without radiation therapy (RT). However, these approaches are based upon large population studies and could be improved for individualized patient care. Preclinical data show that combining chemotherapeutic agents with low-dose RT could result in tumor suppression due to increased apoptotic signaling, reactive oxygen species (ROS), and DNA damage (12). Currently, there are no studies that analyze the effects of chemotherapy combined with RT on the markers expressed in these changes (13). Evaluating these effects in robust pre-clinical models, such as 3D tumor organoids, may support and unlock insights for individualized care.

This study aims to evaluate the therapeutic potential of combination chemotherapy and radiation therapy using pancreatic tumor organoids (PTOs) derived from murine Panc02 tumors. To evaluate tumor microenvironment fidelity in these PTOs, the expression of two key TME markers, alpha-smooth muscle actin (α-SMA) and vimentin, was assessed using immunohistochemical techniques. PTOs were then treated with single and combination therapies to compare treatment responses. Finally, the effects on ROS and γH2AX were assessed using immunofluorescence.

**Figure 1:**
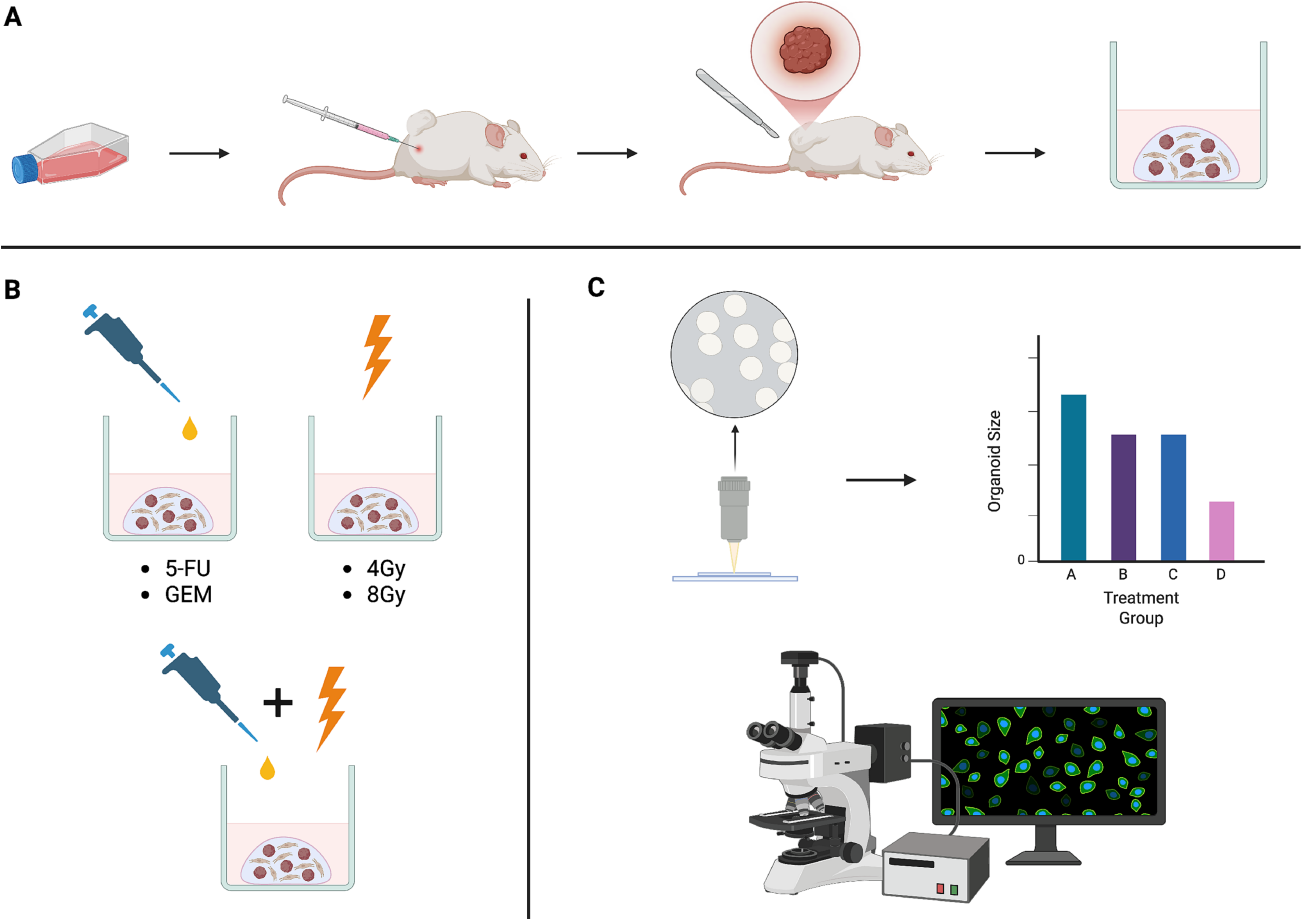
**(A)** Panc02 cells were subcutaneously injected into the flanks of mice and subsequently resected to generate tumor organoids of diverse cell types. **(B)** Tumor organoids were treated with chemotherapy drugs often used in standard-of-care regimens, ionizing radiation, and combinations of chemotherapy and radiation. **(C)** Treatment response was assessed both qualitatively and quantitatively using brightfield imaging and immunofluorescent techniques. Schematic created in BioRender.

While some studies have manipulated the expression of specific markers to sensitize pancreatic cancer models to chemotherapy (14), this study evaluated the direct impact of chemotherapy on markers such as ROS and γH2AX. By assessing the efficacy of combining chemotherapy with RT and validating ROS-induced DNA damage as a potential mechanism of PTO inhibition, this study highlights the effects of the combination approach to treatment and the importance of the organoid model as a promising platform for precision medicine in pancreatic cancer (see Figure 1).

## Methods

### Mouse pancreatic tumor organoid culture

The mouse pancreatic tumor organoids were derived from flank tumors developed in c57bl/6 mice following subcutaneous injection of Panc02 cells. Organoids were grown and cultured in Matrigel^®^ domes on 24-well plates, as described previously (3).

### Immunofluorescent and immunohistochemical staining of mouse pancreas tumor organoids and tumor tissue

The mouse pancreatic tumor organoids were generated and propagated in Matrigel^®^ domes in 24-well plates as described above. Immunofluorescence staining was performed as follows: the organoids were fixed in formalin tissue fixation buffer solution with 10% neutral buffer (Sigma Chemical, United States). Before staining, the fixative was removed, and organoids were washed with 1× PBS three times for 5 min each. Organoids were then permeabilized with 0.2% Triton X-100 for 5 min, washed with 1× PBS for 5 min, and blocked with the blocking buffer (Vector Labs, United States) for 1 h. Primary antibodies used were α-SMA (smooth muscle actin): rabbit polyclonal unconjugated 1:100 dilution (ABclonal antibody, #A7248, United States), vimentin: Alexa Fluor 594 conjugated anti-mouse vimentin 1:300 dilution (BioLegend, #699303, United States), and γ-H2AX: phospho gamma S139 H2AX-anti-rabbit (Cell Signaling Technology, #9718S, United States) 1:400 dilution. Incubation with primary antibody was performed overnight in a 1:5 diluted blocking buffer in a 4 °C cold room. Next, the primary antibodies were removed and washed as described above. For the unconjugated α-SMA and γ-H2AX detection, Alexa Fluor 488 rabbit secondary antibody (Thermo Fisher Scientific, #A11008, United States) at 1:300 dilution was used for 1 h. Hoechst 33342 dye (Thermo Fisher Scientific, #H21492, United States) with a concentration of 1 μg/mL was then added for 5–10 min. The antibody and Hoechst dye were then discarded, and the organoids were washed in the same way as described earlier. Finally, the organoids were kept in 1× PBS, and images were captured using an EVOS fluorescence microscope at 20x magnification (Thermo Fisher Scientific, United States). For staining, slides were deparaffinized, unmasked (Vector Labs, United States), and blocked with blocking buffer (Vector Labs, United States). Staining with the α-SMA primary antibody was the same as described above. For the secondary antibody, Vectastain Universal Quick Kit was used according to the manufacturer’s instructions. Finally, ImmPACT DAB (Vecta Stain, United States) was used to develop a brown color. After washing with water, the slides were air-dried and mounted with cytoseal-60 (Epredia, United States). The images were captured with an EVOS Xl color microscope.

### Treatment of organoids with chemotherapy drugs, radiation, and brightfield imaging

The organoids were grown in 25 μL Matrigel^®^ domes on 24-well plates with 500 μL of organoid growth media. Treatment groups included 5-FU (0–100 μM), gemcitabine (5 μM), RT (4 Gy or 8 Gy), and 5-FU or gemcitabine + RT. For the single-modality groups, treatment was administered once, and organoids were allowed to grow for 5 days. For the combination groups, RT was administered 24 h after the initiation of chemotherapy treatment, and organoids were allowed to grow for an additional 4 days. At the end of the treatment, images were captured with an EVOS light microscope and fixed in formalin, as described above. After removal of the fixative, the organoids were washed with 1x PBS, three times each for 5 min, permeabilized with 0.2% Triton X-100 for 5 min, and stained with Hoechst dye (1 μg/mL) for 10 min. The images were captured with an EVOS fluorescence microscope (blue filter).

### Measurement of ROS

Organoids were grown and treated as above. At the end of radiation treatment, organoids were incubated with ROS substrate DHE (dihydroethidium, Medchem Express, United States) 20 μg/mL in growth media and incubated for approximately 2 h. Images were captured with the EVOS fluorescence microscope (red filter). Brightfield images were also obtained.

### Tumor organoid response and statistical analysis

Tumor organoid response after treatment was assessed by averaging the size of the five largest organoids, as determined by ImageJ. The organoids that did not receive treatment were considered a control group, and all groups’ average sizes were normalized to the average size of the control. The responses of the groups were directly compared using a one-sided Student’s *t*-test, with a particular focus on comparing groups receiving combination treatments to those receiving the most effective individual component of those combinations. Differences in the average organoid size were considered significant with a *p*-value of <0.05.

## Results

### Characterization of mouse pancreas tumor organoids as a model to study chemotherapy drugs and radiation treatment response

**Figure 2:**
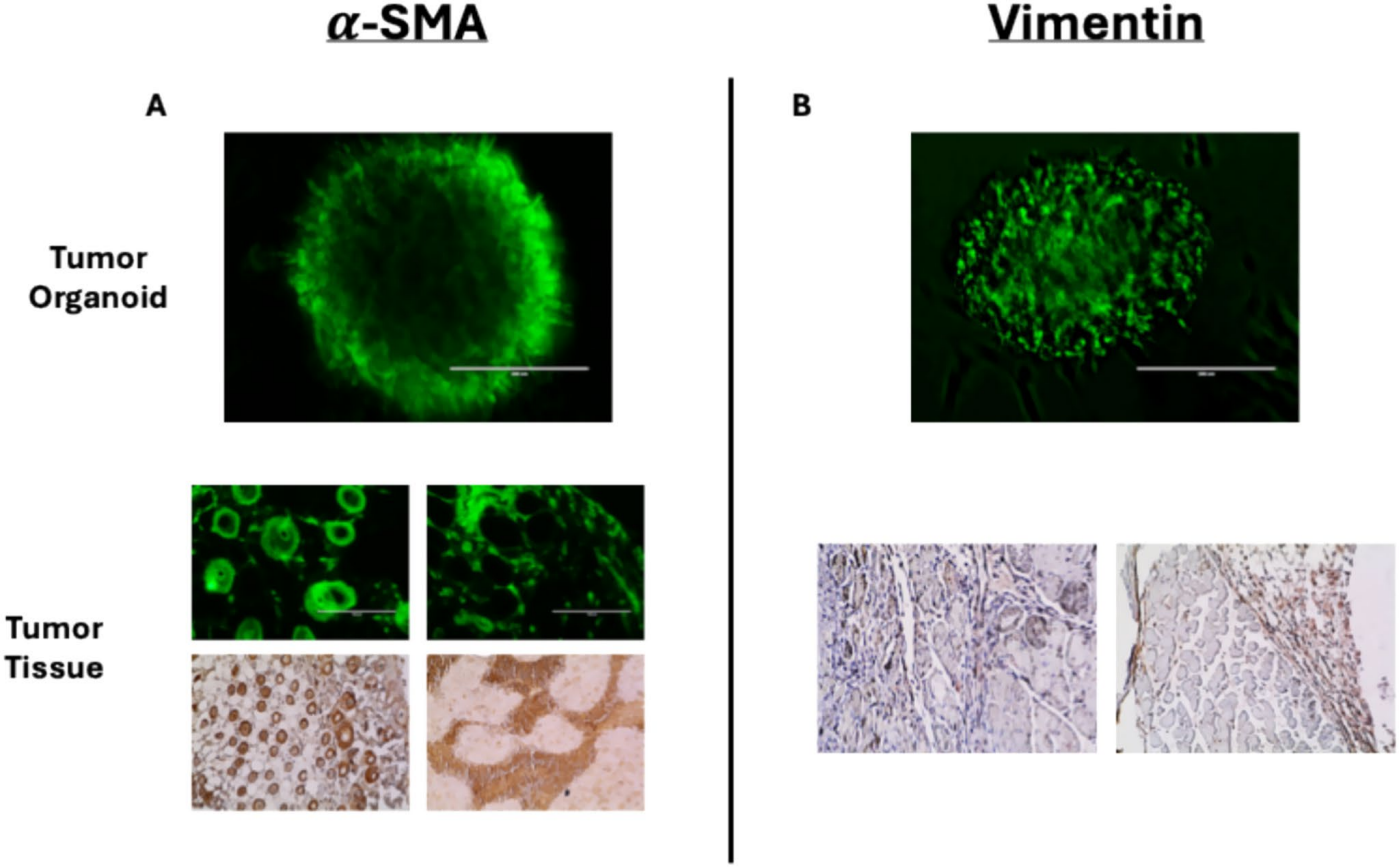
Immunofluorescent staining of tumor organoids and immunohistochemical staining of mouse-derived pancreas tumor tissues for **(A)** α-SMA and **(B)** vimentin. Immunofluorescent stains of tumor tissues for α-SMA corroborate the DAB stain.

It has been shown that mouse PTOs, derived from pancreas tumor tissues or patient-derived xenografts, closely mimic many of the histological, genetic, and phenotypic features of PDAC (9, 10). In this study, we have observed that tumor organoids may maintain the key characteristics of the original TME, expressing α-SMA and vimentin in patterns similar to tumor tissue (Figure 2). Thus, these tumor organoids represent cellular heterogeneity, tumor architecture, and stromal interactions with other important cell types, including but not limited to cancer-associated fibroblasts (CAFs).

### Treatment response of mouse pancreas tumor organoids to 5-FU, gemcitabine, and radiation alone and in combination

**Figure 3:**
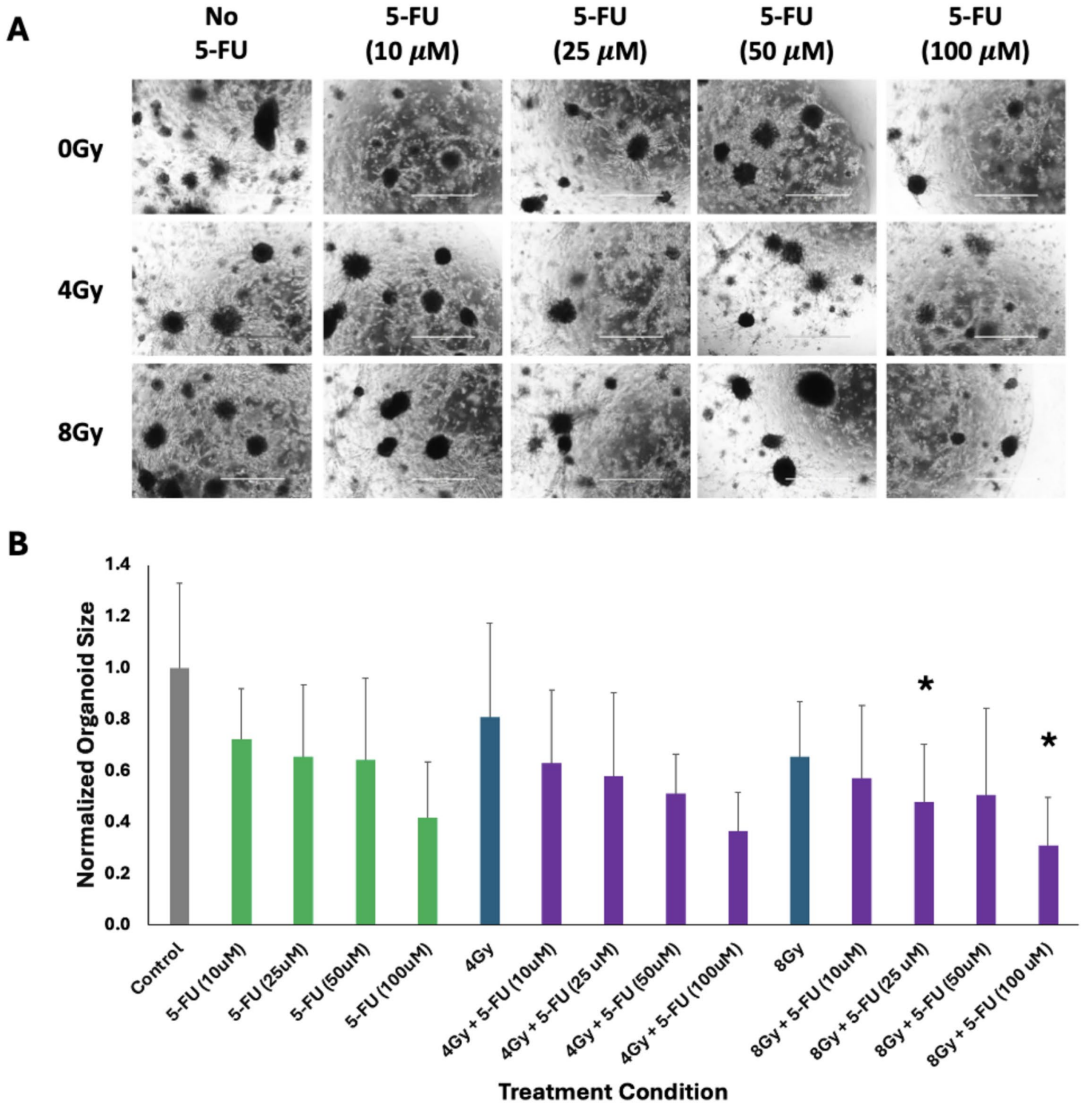
Responses of pancreas tumor organoids to treatment with 5-FU (10–100 μM), RT (4 and 8 Gy), and 5-FU + RT at various doses and combinations. The scale bar at the bottom right of each image represents 1,000 μm. **(A)** Brightfield imaging 5 days after the initiation of treatment. **(B)** Average size of the five largest organoids in each of the above images, normalized to the control. Green bars represent those organoids treated with only 5-FU, blue bars represent those treated with only RT, and purple bars represent those treated with a combination of 5-FU and RT. The asterisk indicates a statistically significant (*p* < 0.05) decrease in average organoid size as compared to the most effective single-modality treatment. Error bars represent 1 standard deviation of values.

The sensitivity of mouse PTOs to different doses of 5-fluorouracil (5-FU), gemcitabine, and radiation as monotherapy and combination therapy was evaluated. Tumor organoids were treated with 10–100 μM of 5-FU and monitored for growth inhibition by brightfield imaging. The data showed dose-dependent growth inhibition. Only 100 μM of 5-FU achieved >50% inhibition of tumor organoids, suggesting that single-modality treatment needs high doses to inhibit tumor organoid growth. Notably, co-treatment of 5-FU with radiation (e.g., 25 μM + 8 Gy) yielded synergistic effects and showed significant growth inhibition (*p* < 0.05) compared to single-modality treatments (Figure 3). Furthermore, we observed >70% growth inhibition in tumor organoids treated with 100 μM of 5-FU combined with 8 Gy of radiation. It is also important to note that the combination of 50 μM 5-FU + 8 Gy RT resulted in a response that was not consistent with a dose-dependent trend, which might be expected given the responses to combinations of 5-FU with 4 Gy RT. This is most likely due to the presence of an outlier organoid, which was approximately 2 standard deviations larger than the average of the other organoids in the group and was the largest in any group receiving 8 Gy of RT. Given the totality of the evidence, it is reasonable to conclude that a combination of low doses of 5-FU + radiation reduced organoid viability more effectively than either treatment alone.

**Figure 4:**
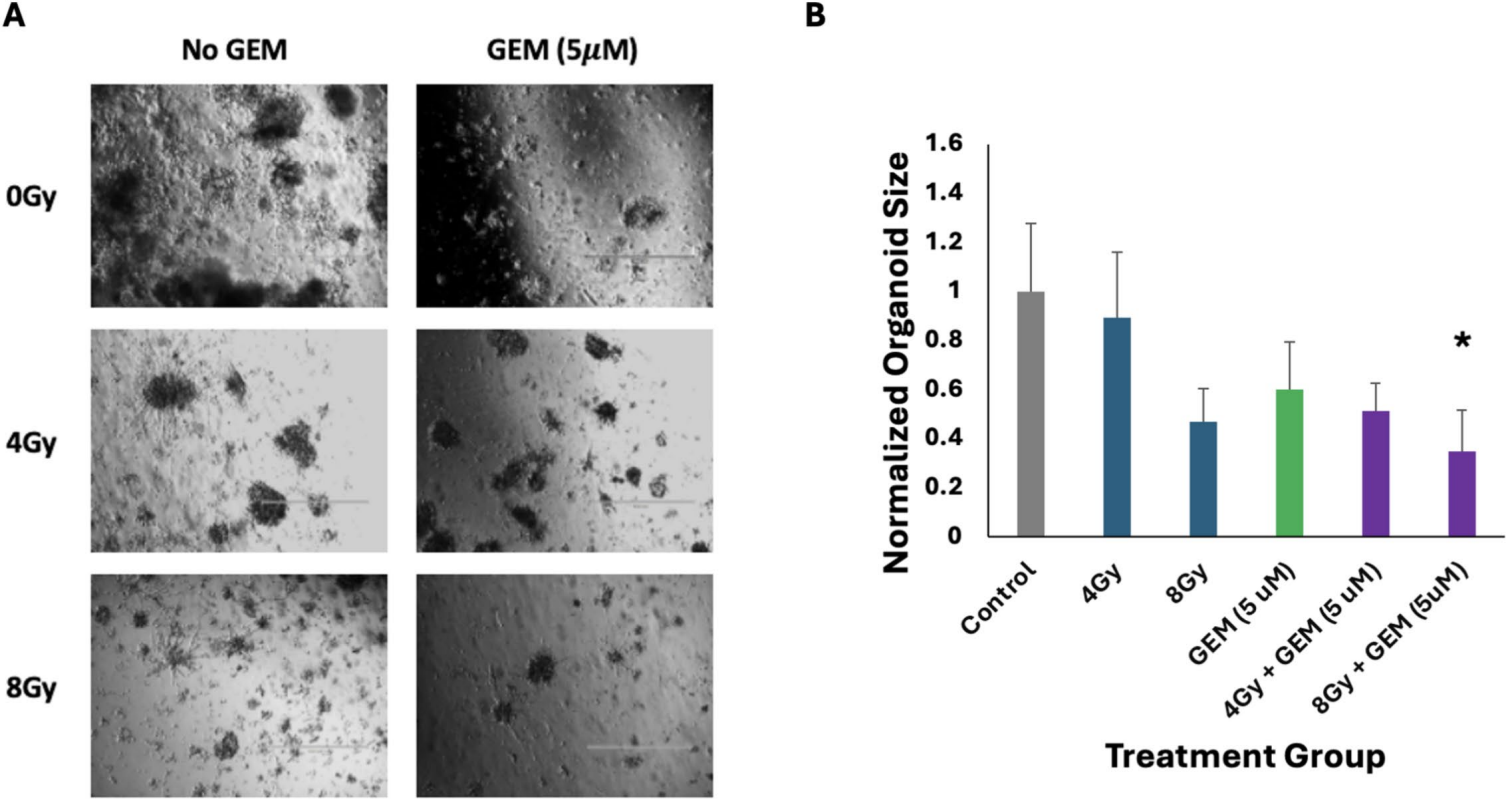
Responses of pancreas tumor organoids to treatment with gemcitabine (GEM, 5 μM), RT (4 and 8 Gy), and GEM + RT. **(A)** Brightfield imaging 5 days after the initiation of treatment. The scale bar at the bottom right of each image represents 1,000 μm. **(B)** Average size of the five largest organoids in each of the above images, normalized to the control. The green bar represents those organoids treated with only GEM, the blue bars represent those treated with only RT, and the purple bars represent those treated with a combination of GEM and RT. The asterisk indicates a statistically significant (*p* < 0.05) decrease in average organoid size as compared to the most effective single-modality treatment. Error bars represent 1 standard deviation of values.

**Figure 5:**
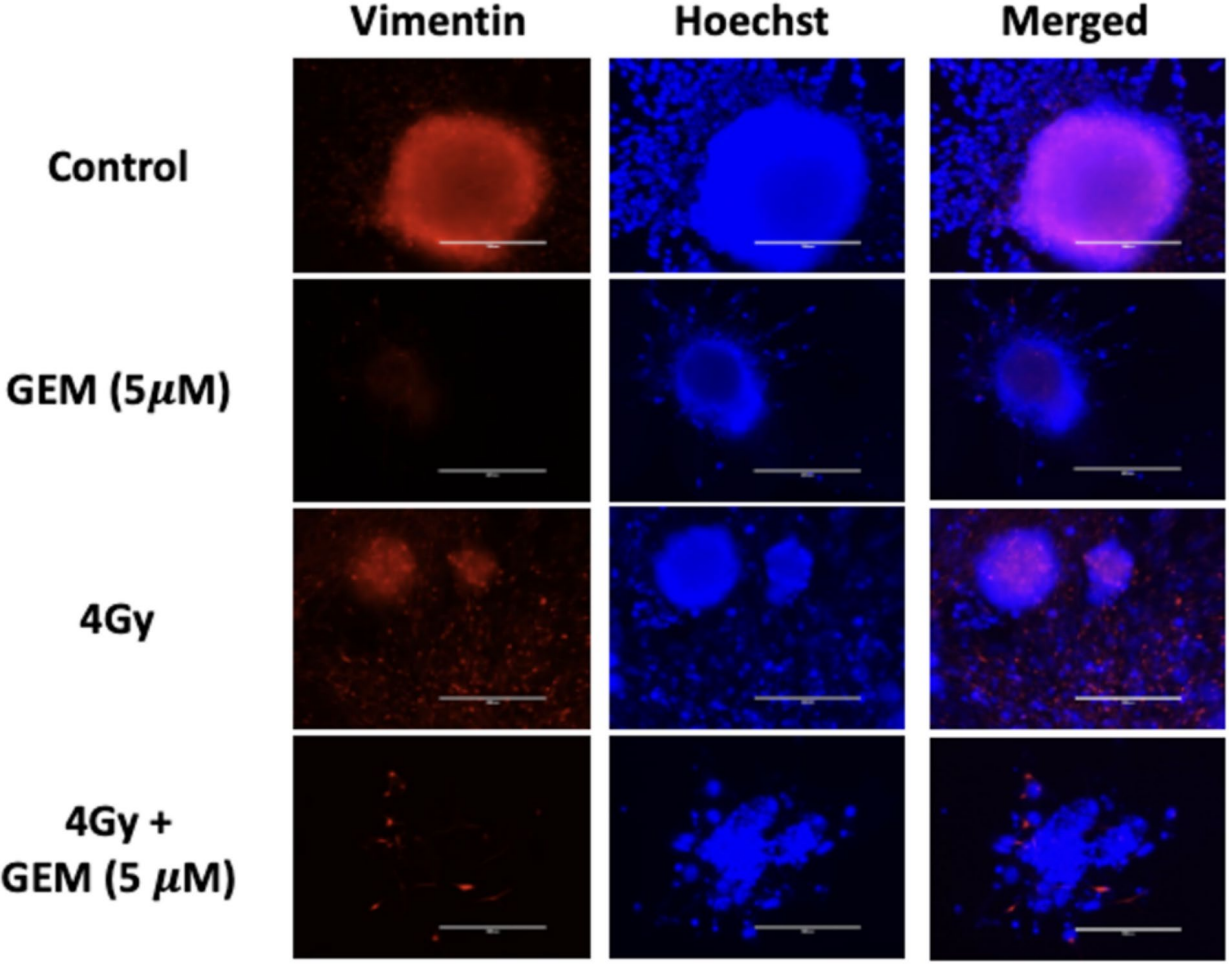
Tumor organoid expression of vimentin (red) with nuclear stain (blue) in response to gemcitabine (GEM, 5 μM) alone, RT (4 Gy) alone, and a combination of gemcitabine and RT at the same doses. The scale bar represents 200 μm.

The response of PTOs to gemcitabine + radiation treatment was also examined. Tumor organoids treated with a low dose of gemcitabine followed by 4 Gy or 8 Gy of radiation showed greater growth inhibition compared to single-modality treatments. There was approximately 50 and 70% growth inhibition in PTOs treated with 4 Gy + 5 μM gemcitabine and 8 Gy + 5 μM gemcitabine, respectively (Figure 4). While the response of the group treated with 4 Gy + 5 μM gemcitabine was not significant compared to the response of the group treated with gemcitabine alone (*p* = 0.10), it was significant when compared to the group treated with 4 Gy alone (*p* < 0.05). Additionally, immunofluorescent staining revealed a disorganized expression of vimentin following treatment with 4 Gy + 5 μM gemcitabine, which was not observed following treatment with either modality in isolation (Figure 5). These data suggest that the combination treatment was more effective, leading to enhanced PTO growth inhibition and TME disruption.

This study demonstrated that pancreatic tumor organoids exhibit distinct responses to individual and combination treatments. Specifically, the combination treatment of low doses of 5-FU or gemcitabine with radiation shows significant inhibition of tumor organoid growth. Therefore, it is reasonable to consider how the combination treatment approach may target different signaling pathways to exert a synergistic effect and overcome therapy resistance.

### The combination of chemotherapy and radiation targets different cellular pathways to induce cell death in tumor organoids

**Figure 6:**
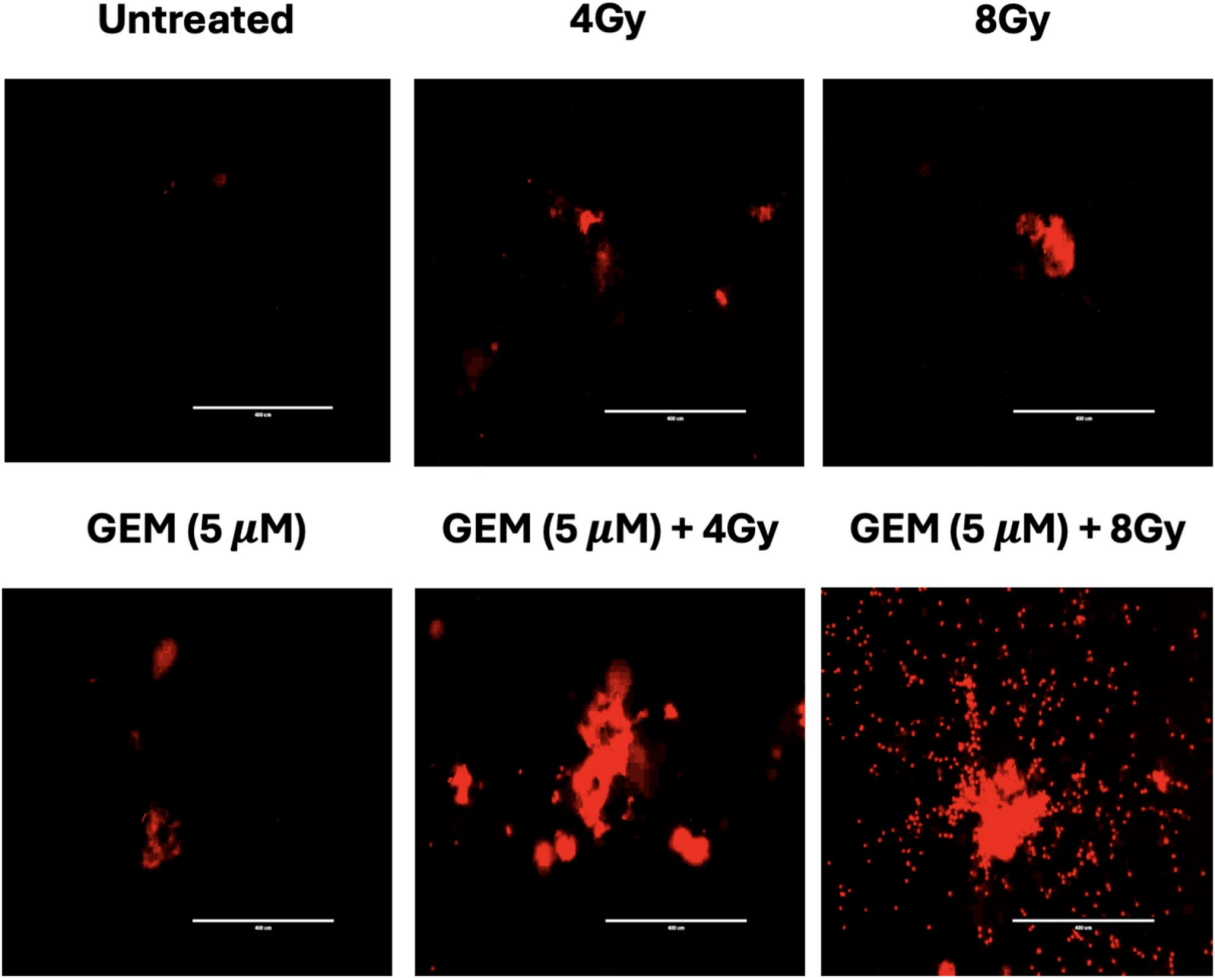
Tumor organoid expression of reactive oxygen species (ROS) after treatment with RT (4 and 8 Gy), gemcitabine (GEM, 5 μM), and combinations of gemcitabine and RT. Each label is above the corresponding image. The scale bar represents 400 μm.

Radiation and chemotherapy drugs such as 5-fluorouracil and gemcitabine each use cytotoxic effects through different mechanisms. When used alone or in combination, these therapies target various cellular signaling pathways, leading to cell death via DNA damage, cell cycle arrest, and replication stress (15). When PTOs were treated with either gemcitabine or radiation, cytotoxic ROS were produced; however, the combination of gemcitabine and radiation appeared to generate a greater amount of ROS (Figure 6).

**Figure 7:**
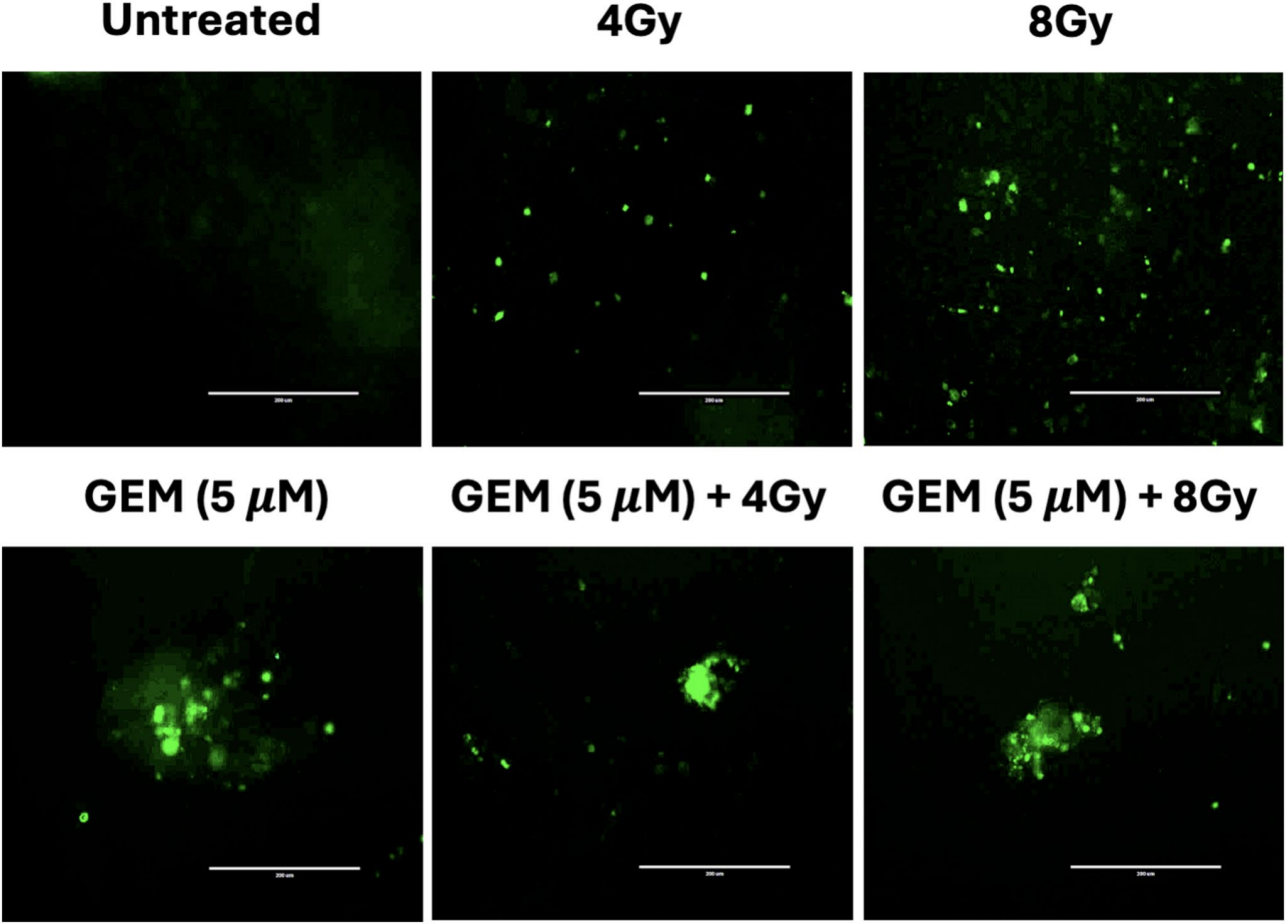
Immunofluorescent staining of phosphorylated γH2AX in tumor organoids after treatment with RT (4 and 8 Gy), gemcitabine (GEM, 5 μM), and combinations of RT and GEM shows evidence of DNA double-strand breaks. The scale bar represents 200 μm.

We also assessed the presence of double-strand breaks (DSBs) in tumor organoids treated with gemcitabine and radiation individually and in combination by staining for γH2AX. After treatment, H2AX was quickly phosphorylated at the site of DSBs, which was detected using immunofluorescence microscopy. The data also demonstrated that gemcitabine acts as a radiosensitizer, increasing radiation-induced DNA DSBs (Figure 7). This finding would be consistent with pancreatic cancer cell line work suggesting that gemcitabine synchronizes cells in the S-phase and inhibits repair pathways, making them more vulnerable to radiation-induced DSBs (16).

Based on these results, we envisage that 5-FU and gemcitabine both reduce the DNA repair ability of cells and, as a result, cause tumor cells to become more sensitive to radiation-induced DNA strand breaks and cell death.

## Discussion

Pancreatic ductal adenocarcinoma continues to be among the most aggressive and lethal malignancies due to its late diagnosis, high metastatic potential, and poor responsiveness to current therapeutic strategies (17). The 2D cancer cell model has long served as a platform to study treatment responses and drug efficacy; however, it has not successfully replicated the complex tumor architecture and tumor microenvironment of patients’ tumors (3, 17). While *in vivo* models offer more physiological relevance, they are time-consuming, resource-intensive, and often differ biologically from human tumors (18). The initiation of 3D tumor organoid cultures, derived from tumor tissues or patient-derived xenografts, offers an advanced *ex vivo* system that maintains the histological, molecular, and functional properties of the parent tumor (19, 20). The organoids utilized in this study showed expression of TME markers α-SMA and vimentin in patterns similar to *in vivo* tissues, indicating that key components of tumor-stromal interaction and heterogeneity are also present in tumor organoids. This makes the organoid platform ideal for mimicking the treatment dynamics in PDAC and for exploring therapeutic responses under translational conditions (6, 21). In this study, mouse pancreas tumor organoids were used as a model system to evaluate the dose effectiveness of 5-fluorouracil and gemcitabine combined with ionizing radiation (22).

The results showed that 5-FU monotherapy produced a dose-dependent inhibition of organoid growth, with the highest dose of 100 μM causing approximately a 50% reduction in organoid size. When low doses of 5-FU were combined with radiation (4 Gy and 8 Gy), the treatment efficacy increased (23–25). Furthermore, combinations of 25 or 100 μM 5-FU with 8 Gy radiation produced significantly greater inhibitory effects than either treatment modality administered alone (*p* < 0.05). The latter combination was most effective and inhibited organoids by >70%. Previous studies have also shown that a combination of low-dose chemotherapy and radiation minimizes the cytotoxic effects in normal tissues (26, 27). This suggests that there exists a synergistic effect where the combination of chemotherapy and radiation causes increased DNA damage and reduced DNA repair capabilities, thereby promoting apoptotic cell death more effectively than monotherapy (28, 29).

Similarly, the efficacy of gemcitabine was enhanced when combined with radiation. Compared to the most effective single modality, a low dose of gemcitabine (5 μM) followed by 4 or 8 Gy radiation led to increased inhibition of tumor organoid growth, by approximately 50% (*p* = 0.10) and 70% (*p* = 0.05), respectively. Furthermore, pre-treatment with gemcitabine exhibited radiosensitizing properties, rendering tumor cells more susceptible to radiation-induced DNA damage and apoptotic cell death (12, 28, 30). As previously postulated through research in pancreatic cancer cell line models, the radiosensitizing effect of gemcitabine likely stems from the inhibition of DNA replication and repair pathways, particularly its action on replication forks and the suppression of DNA repair machinery. This effect is especially pronounced when cells are synchronized in the S-phase, thereby amplifying the cytotoxic impact of ionizing radiation (31, 32).

To better understand the mechanistic effects of combining chemotherapy with radiation, we examined the cellular pathways influenced by the combination of treatments. The data suggest that gemcitabine, in combination with radiation, increases intracellular ROS (Figure 6), an important mediator of oxidative DNA damage, mitochondrial dysfunction, and apoptosis (13, 33). Thus, a synergistic enhancement of ROS may be a component of the mechanism underlying the combination effect. Additionally, there is evidence suggesting that combination therapies may activate tumor suppressor pathways such as p53 and checkpoint kinases (Chk1/Chk2), triggering irreversible cell cycle arrest and caspase-mediated apoptosis (34).

The results showed that the combination treatment also increased γH2AX, a marker indicative of DNA double-strand breaks (DSBs). The γH2AX foci formation was of notably high intensity following treatment with gemcitabine or a combination of gemcitabine and radiation, providing evidence of elevated DNA damage in tumor organoids (Figure 7). As a sensitive and quantifiable biomarker, γH2AX serves not only as a marker of therapeutic efficacy but also as a potential tool for optimizing treatment timing and dosage in future clinical applications (35, 36). Pretreatment with gemcitabine may further enhance radiosensitivity by synchronizing cells in a vulnerable phase of the cell cycle and preventing the repair of radiation-induced DSBs.

Collectively, these findings support the use of pancreas tumor organoids as a robust and physiologically relevant model for deriving mechanistic and translatable insights regarding therapeutic approaches. The notably enhanced responses of tumor organoids to combination treatments underscore the potential of multi-modal therapies in overcoming treatment resistance (37, 38). Future studies should aim to identify molecular predictors of treatment response and resistance using patient-derived organoid models, paving the way for the development of more effective, personalized therapeutic strategies.

## Data Availability

The original contributions presented in the study are included in the article/supplementary material, further inquiries can be directed to the corresponding authors.
